# Harnessing the power of natural alkaloids: the emergent role in epilepsy therapy

**DOI:** 10.3389/fphar.2024.1418555

**Published:** 2024-06-19

**Authors:** Siyu Li, Xinyu Lin, Lijuan Duan

**Affiliations:** ^1^ Department of Neurosurgery, Clinical Trial Center, West China School of Nursing, West China Hospital, Sichuan University, Chengdu, China; ^2^ School of Pharmacy, Chengdu University of Traditional Chinese Medicine, Chengdu, China

**Keywords:** epilepsy, natural alkaloid, antiseizure medications, neuropharmacological mechanism, nanotechnology, gaba receptor

## Abstract

The quest for effective epilepsy treatments has spotlighted natural alkaloids due to their broad neuropharmacological effects. This review provides a comprehensive analysis of the antiseizure properties of various natural compounds, with an emphasis on their mechanisms of action and potential therapeutic benefits. Our findings reveal that bioactive substances such as indole, quinoline, terpenoid, and pyridine alkaloids confer medicinal benefits by modulating synaptic interactions, restoring neuronal balance, and mitigating neuroinflammation—key factors in managing epileptic seizures. Notably, these compounds enhance GABAergic neurotransmission, diminish excitatory glutamatergic activities, particularly at NMDA receptors, and suppress proinflammatory pathways. A significant focus is placed on the strategic use of nanoparticle delivery systems to improve the solubility, stability, and bioavailability of these alkaloids, which helps overcome the challenges associated with crossing the blood-brain barrier (BBB). The review concludes with a prospective outlook on integrating these bioactive substances into epilepsy treatment regimes, advocating for extensive research to confirm their efficacy and safety. Advancing the bioavailability of alkaloids and rigorously assessing their toxicological profiles are essential to fully leverage the therapeutic potential of these compounds in clinical settings.

## Graphical Abstract

**Figure:**
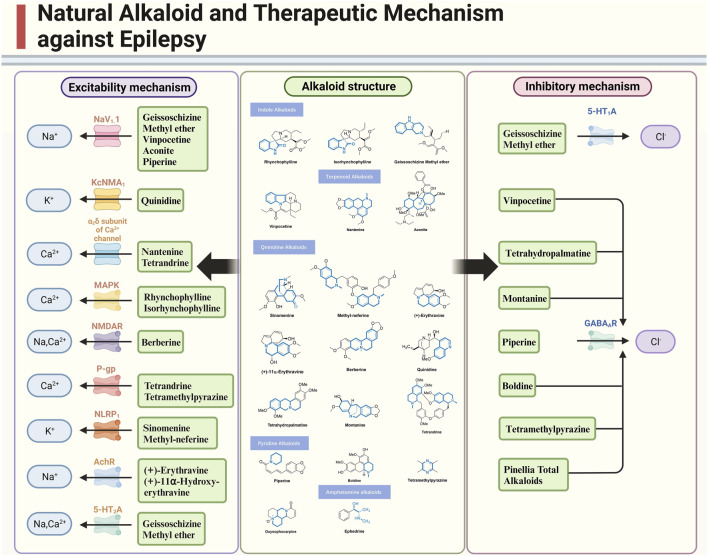
Therapeutic mechanisms of natural alkaloids in epilepsy treatment.

## 1 Introduction

**FIGURE 1:**
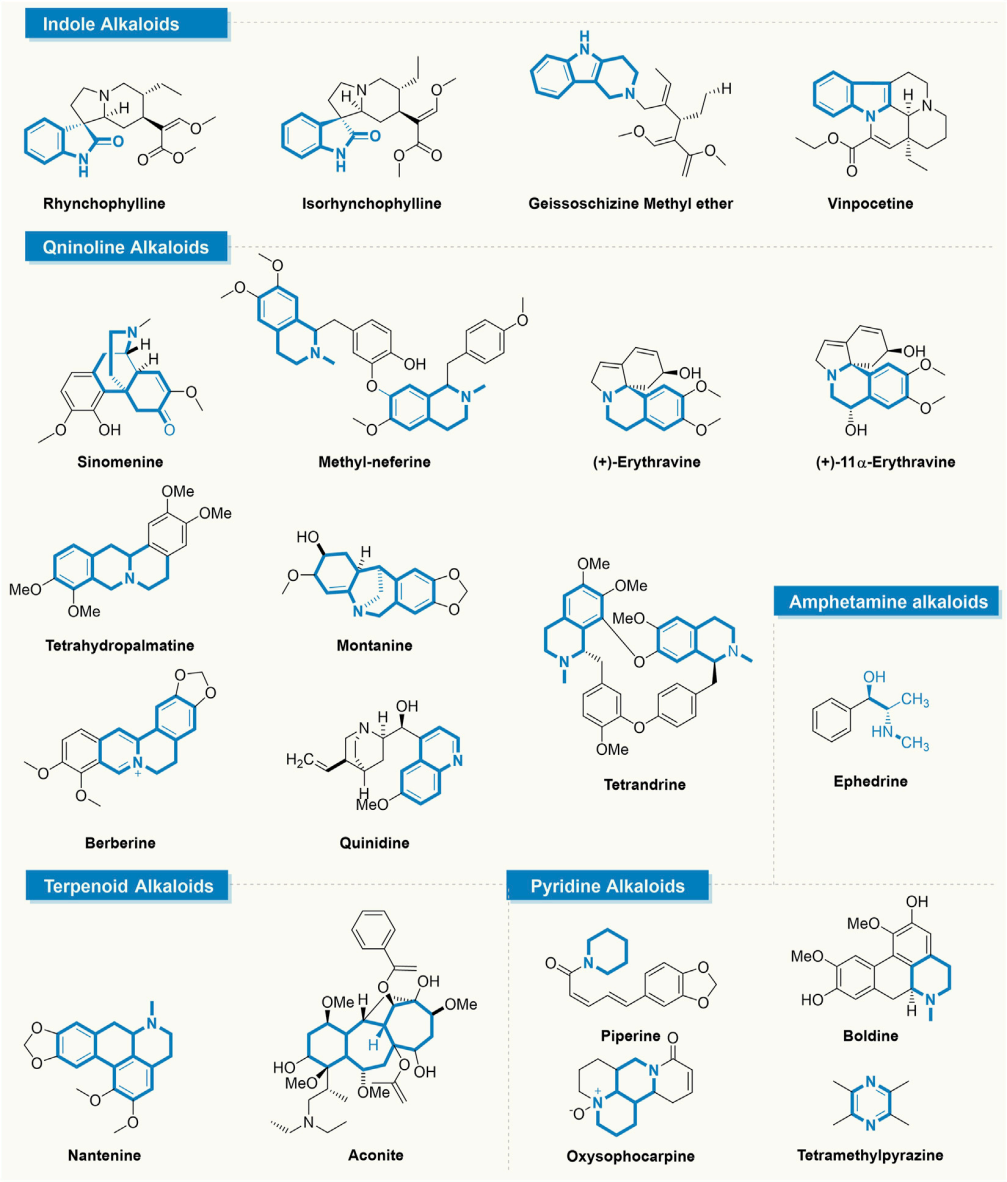
Natural alkaloids for the treatment of epilepsy.

Epilepsy, often metaphorically described as the “goat disease,” is a chronic neurological ailment marked by frequent seizures stemming from abrupt and intense neuronal electrical activity (He et al., 2021). These recurrent episodes not only cause immediate neurological damage but also lead to persistent cognitive impairments, psychiatric conditions, and other lasting neural consequences, significantly affecting the patients’ quality of life (Wu et al., 2023). This condition afflicts approximately 65 million individuals worldwide, with a higher incidence in the developing regions, where up to 80% of these individuals are found (Kanner and Bicchi, 2022). Traditional antiseizure medications (ASM), including phenytoin and lamotrigine, though commonly prescribed, often fail to provide sufficient efficacy and are poorly tolerated by patients (Figure 1) (Ali et al., 2022). Recent advancements in treatment options, like brivaracetam and cannabidiol, offer increased therapeutic potential and better tolerability, especially beneficial for those suffering from resistant variants such as temporal lobe epilepsy. Nonetheless, a significant proportion of patients, estimated at 30%–40%, exhibit resistance to these drugs (Bahr et al., 2019), and the majority of conventional therapies do not effectively halt the disease’s progression, often resulting in cognitive deterioration and impacting adherence to treatment regimes. Consequently, the pursuit of innovative, potent, and accessible antiseizure methods remains an urgent and critical objective in addressing this pervasive health issue.

In light of the inadequacies of existing antiseizure medications, recent scientific inquiry has turned to the possibilities offered by phytochemicals (Akkol et al., 2021). This trend is marked by a noticeable inclination towards utilizing plant-based natural substances in drug development (Zhao et al., 2018). The advantages of these substances are highlighted not only by their potent biological activities but also by the relative ease of their extraction and the broad possibilities for exploring these biogenic materials. This makes them particularly appealing and promising alternatives to synthetic drugs in pharmaceutical research (Challal et al., 2023). Investigative efforts have uncovered structural similarities between conventional small-molecule antiseizures and natural alkaloids, which are nitrogen-containing organic compounds commonly present in plants. These alkaloids have been historically recognized for their wide-ranging pharmacological effects, including neuroprotective actions, reduction of neural hyperexcitability, and anticonvulsant benefits (Bui et al., 2023). Therefore, alkaloids are increasingly becoming focal in the innovation of antiseizure drugs (AED). Furthermore, empirical research substantiates that approximately 80% of epilepsy patients in the developing world rely on traditional plant-based treatments, a practice now receiving growing support through extensive pharmacological studies (Kakooza-Mwesige, 2015). The inherently low toxicity and potent antiseizure efficacy of certain plants and their alkaloid components mark them as crucial in the search for innovative AED.

**FIGURE 2:**
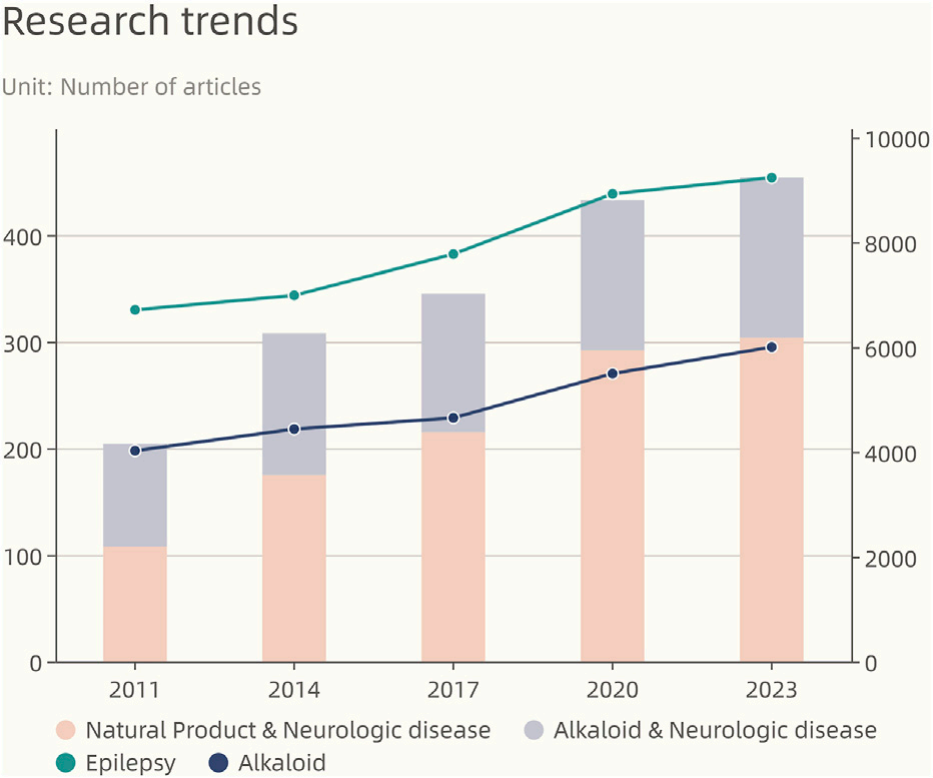
Research progress of natural products and alkaloids in the treatment of neurological diseases, as well as the trend of publishing articles on epilepsy and alkaloids in the past years (All data from web of science).

**FIGURE 3:**
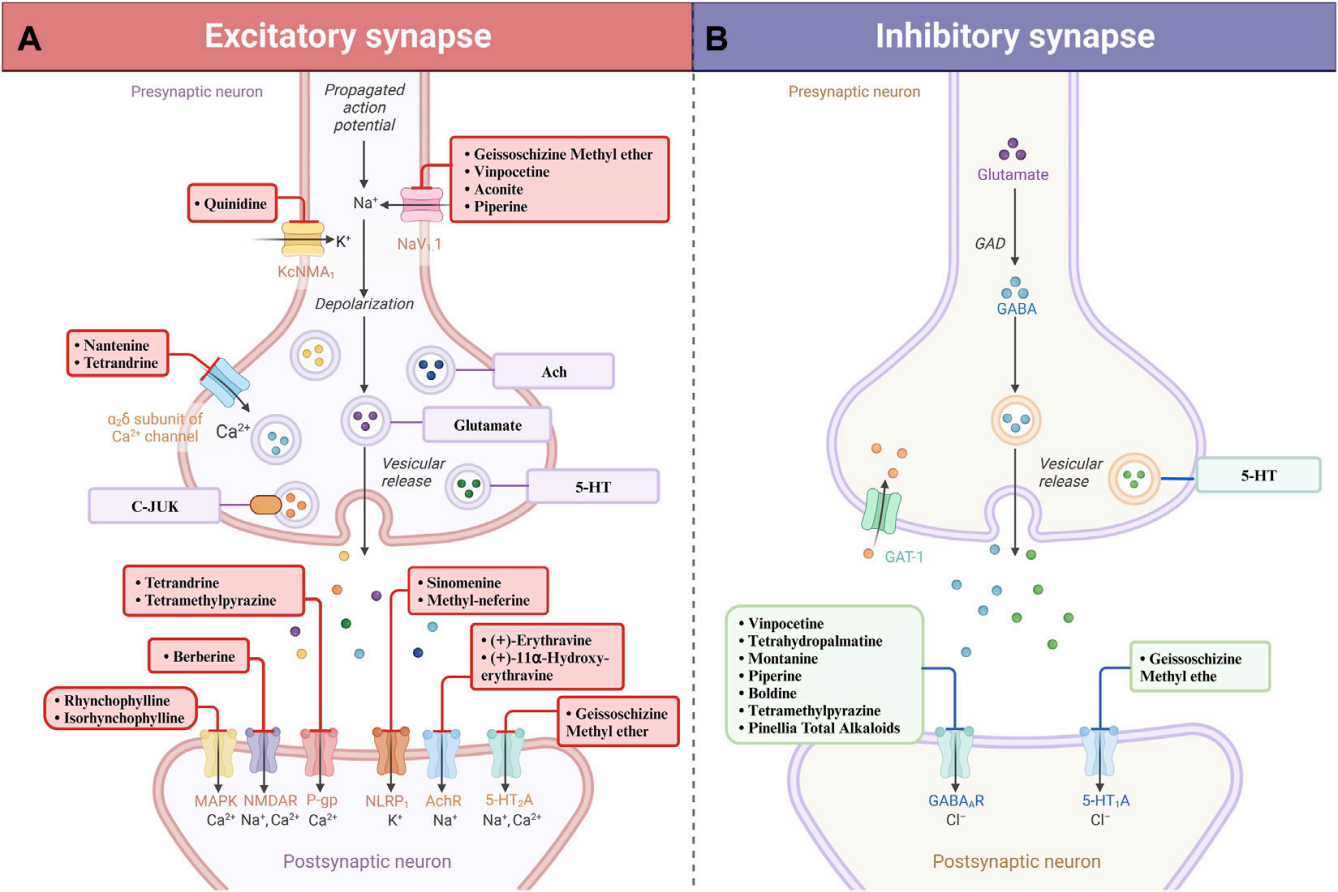
Natural alkaloids exert their therapeutic effects in the treatment of epilepsy by agonizing excitatory receptors **(A)** and agonistically activating inhibitory receptors **(B)**.

Historical use of botanicals like St. John’s wort, ginkgo, and garlic, recognized for their antiseizure qualities (Aghdash, 2021), and a variety of bioactive alkaloids, such as indole, quinoline, and pyridine alkaloids, have been identified with proven antiseizure effects (Figure 1). These alkaloids’ influence on neurotransmitter receptors and ion channels offers a promising framework for creating advanced antiseizure treatments. In recent years, there has been a notable surge in the development of plant-derived AED, underscoring the untapped possibilities of phytochemicals in this area of therapy (Figure 2) (He et al., 2021). It is important to highlight the growing relevance of alkaloids in treating neurological disorders. However, in comparison to other natural antiseizure agents, these advancements are not as widely recognized. With this in mind, our review seeks to amalgamate the latest developments in alkaloid-based antiseizure treatments and to stimulate more targeted research in this domain (Figure 3; Table 1). Utilizing these novel compounds to expand the repertoire of antiseizure alkaloids has the potential not only to enhance the treatment efficacy for patients with intractable epilepsy but also to shed light on the etiological aspects of the condition, thereby contributing to better understanding and improvement of clinical management approaches.

**TABLE 1:** Natural alkaloids utilized in epilepsy treatment through regulation of synaptic function and neurotransmitter release.

Alkaloid type	Natural source	Compounds	Animal models	Mechanisms	Test dose	Ref.
*Indole alkaloids*	*Uncariarhynchophylla (Miq.) Miq. ex Havil.*	Rhynchophylline Isorhynchophylline	Marine alginic acid in mice	Inhibition of NMDA receptor current Stimulated phosphorylation of c-JunN terminal kinase within the MAPK pathway Woke MAPK mediated signal transduction Inhibition of pro-inflammatory cytokine IL-1β expression of brain derived neurotrophic factor genes Downregulated the expression of N-methyl-D-aspartate receptor subunit 2B Reduced mitochondrial respiratory activity and inhibited ROS production	1–30 μM	He et al., 2021, Hu et al. (2018), Geng et al. (2021), Mathien et al. (2021), Ho et al. (2014), Zhou et al. (2010), Ndagijimana et al. (2013)
	*Uncariarhynchophylla (Miq.) Miq. ex Havil.*	Geissoschizine Methyl ether	MES in mice	Suppressed voltage gated sodium (NaV), calcium (CaV) and delayed rectifier potassium (IK) currents Regulated serotonin activity, antagonized 5-HT2A, 5-HT2C, and 5-HT7 receptors	20 mg	Jiang et al. (2015), Lai et al. (2014), Xie et al. (2020), Nakamura et al. (2018), Marucci et al. (2021)
	*Vinca minor L.*	Vinpocetine	Patients with GABRB3 mutations	Improved GABA_A receptor function Inhibited sodium ion and calcium ion conductivity Reduced glutamate release and counteracted glutamate induced depolarization reaction	40 mg	Feng et al. (2017), Meador et al. (2021), Billakota et al. (2019), Gjerulfsen et al. (2023), EL-Sayed et al. (2021), Gómez et al. (2014)
*Quinoline alkaloids*	*Sinomenium acutum* (Thunb.) Rehder & E. H. Wilson	Sinomenine	Pentylenetetrazole in rodents	Mediated inhibition of NLRP_1_ inflammasome Protected hippocampal neurons from PTZ induced damage	20, 40, 80 mg/kg	Nees ex Steud. Li et al. (2023), Hong et al. (2022), Gao et al. (2018), Ramazi et al. (2020)
	*Nelumbo nucifera* Gaertn	Methyl-neferine	Marine alginic acid in rats	Inhibition mediated by NLRP_3_ inflammasome Reduced interleukin-1β; Interleukin-6 and TNF-α level Stabilized glutamate levels and maintained neuronal integrity	10, 50 mg/kg	Liu et al. (2023), Bishayee et al. (2022), Lin et al. (2022b)
	Xanthoxylonclava	Berberine	Marine alginic in mice	Helped correct the expression of STX1B gene Antagonism of NMDA receptors	10, 20 mg/kg	Song et al. (2020), Gawel et al. (2020), Sedaghat et al. (2017), Jiang et al. (2015), Cortes et al. (2015), Hussain et al. (2018)
	*Erythrina mulungu*	(+)-erythravine (+)-11α-hydroxy-erythravine	Pilocarpine in mice	Decreased acetylcholine induced current, antagonism of neuronal nicotinic acetylcholine receptors	1–3 μg/μL	Faggion et al. (2011), Gelfuso et al. (2020), dos Reis et al. (2021), Dakic et al. (2016)
	*Corydalisyanhusuo* W. T. Wang	Tetrahydropalmatine	Marine alginic acid in mice	Reduced dopamine secretion Enhanced GABAergic and cholinergic receptor functions	10–15 mg/kg	Oh et al., 2010, Wu et al. (2015), Chang and Lin (2001)
	*Hippeastrum vittatum* (L’Hér.) Herb.	Montanine	Camphene chlorothiazide in mice	Optimized GABA receptor conduction Corrected abnormal distribution of neurotransmitters	30, 60 mg/kg	Farinon et al. (2017)
	*Stephania tetrandra* S. Moore	Tetrandrine	Camphene chlorothiazide in mice	Reduced P-gp expression at mRNA and protein capacities Adjusted calcium plasma channels	2 μM	Zhang et al. (2018b), Chen et al. (2015)
	*Cinchona ledgeriana* (Howard) Moens ex Trim.	Quinidine	Patients with KCNT_1_ mutations	Treated KCNT_1_ gene mutation Reduced potassium ion channel activity	2 mg/kg	Yoshitomi et al. (2019)
*Terpenoid alkaloids*	*Nandina domestica* Thunb.	Nantenine	Marine alginic in mice	Regulated the inward flow of calcium ions Inhibited the excessive excitability of neurons	20–75 mg/kg	Qin et al. (2021), Steinlein (2014)
	*Aconitum carmichaeli* Debx.	Aconite	Marine alginic in mice	Inhibited the excessive excitability of neurons Stimulated the vagus nerve Controlled sodium ion gated channels	0.1, 1 μM	Ono et al., 2013, Ameri (1998)
*Pyridine alkaloids*	*Piper longum* L.	Piperine	Marine alginic in mice	Increased levels of serotonin and GABA in the cortex and hippocampus, regulated the central neurotransmitter environment Obstructed the function of sodium and calcium channels	2–20 mg/kg	Ren et al. (2019), da Cruz et al. (2013), Mishra et al. (2015)
	*Litsea cubeba* (Lour.) Pers.	Boldine	Pentylenetetrazole in rats	Mutated GABA receptors with protective dysfunction Increased levels of glutathione and superoxide dismutase, with antioxidant capacity	10–75 mg/kg	Moezi et al. (2019), de Souza et al. (2022), Akotkar et al. (2023)
	*Ligusticum sinense*	Tetramethylpyrazine	Marine alginic in mice	Regulated neurotransmitters and inflammatory cytokines, reduced neuronal overactivity, and provided neuroprotection Downregulated P-gp expression in brain tissue Inhibited glutamate synthesis while increasing GABA production and inhibiting neuronal overexcitement Reshaped synapses, upregulated neuroprotective molecules, and reduced apoptosis related factors	20, 50 mg/kg	Danduga et al. (2018), Jin et al. (2019), Meng et al. (2021), Lin et al. (2022a), Lin et al. (2022b), Qian et al. (2023)
	*Sophra flavescens* Ait.	Oxysophocarpine	Camphene chlorothiazide in mice	Reduced the wear and degraded hippocampal neurons Increased antioxidant enzyme activity Inhibited neuronal apoptosis Reduced the expression of pro apoptotic proteins (especially Bax and Caspase-3) Increased the expression of anti-apoptotic protein Bcl-2	40, 80 mg/kg	Xu et al. (2013), Liu et al. (2017)
*Amphetamine alkaloid*	*Pinellia ternata* (Thunb.) Ten. ex Breitenb.	Ephedrine	Pentylenetetrazole in mice	Inhibited the enhancement of GABA energy system function Affected GAD65, GAT-1, GABA-T, and GABA_A_R subunit α4, α5, γ2 and δ expression	400, 800 mg/kg	Deng et al. (2020), Bai et al. (2022)

## 2 Therapeutic promise of natural alkaloids against epilepsy

### 2.1 Indole alkaloids: multipath way modulation and therapeutic potential in epilepsy

Indole alkaloids, a varied class of natural tryptophan derivatives, are recognized for their intricate molecular structures and profound pharmacological effects. These substances are predominantly found in the plant families Oleaceae Hoffmans. and Rubiaceae Juss, exhibiting a range of structures from simple indoles to complex bisindoles (Teng et al., 2023). In the field of antiseizure pharmacotherapy, indole alkaloids target a range of molecular receptors, effectively regulating neurotransmitter movement and ion channel activities. They mitigate epileptic disruptions by reducing excessive Ca^2+^ ion flow through overactive NMDA receptors and by sustaining the suppressive action of GABAergic signaling (Pandey, 2023). Furthermore, these alkaloids influence voltage-gated ion channels (Na^+^, Ca^2+^, and K^+^), curbing the irregular electrical activity that leads to seizures. The diverse pathway engagement of indole alkaloids accounts for their comprehensive effectiveness in maintaining the stability of neural network excitability. The detailed exploration of their specific roles in epilepsy treatment is outlined in the following sections (Munir et al., 2020).

Progressing in our discussion, rhynchophylline (RP), an indole alkaloid derived from *Uncariarhynchophylla* (Miq.) Miq. ex Havil of the Rubiaceae Juss family, showcases a broad range of therapeutic properties, encompassing anti-inflammatory effects and neurotransmitter modulation (Hu et al., 2018). This alkaloid has shown effectiveness in treating conditions such as cerebral ischemia, hypoxia, epilepsy, and anxiety. Its active components, notably RP and isocorynoxeine, interact with various signaling pathways and therapeutic targets (Geng et al., 2021), providing neuroprotection through antioxidant activities and neurotransmitter regulation. Experimental studies have demonstrated that RP and isocorynoxeine stimulate the phosphorylation of c-Jun N-terminal kinase in the mitogen-activated protein kinase (MAPK) pathway. This interaction significantly reduces MAPK-mediated signal transduction, thereby lessening neuronal damage in rodent models subjected to kainic acid (KA)-induced acute epileptic seizures (Mathien et al., 2021). Additionally, RP has been observed to reduce neuronal apoptosis in the hippocampus and decrease seizure frequency, influencing the SIRT1/p53/caspase-3 axis. The anticonvulsant effects of RP and isocorynoxeine in KA-exposed rats are further attributed to their regulatory impact on toll-like receptor and neurotrophin signaling pathways, effectively inhibiting the expression of pro-inflammatory cytokines such as IL-1β and genes related to brain-derived neurotrophic factor (Ho et al., 2014). On one aspect, molecules like isocorynoxeine have been shown to decrease NMDA receptor currents, suppress neurotransmitter transmission, and alleviate neural hyperactivity in the brain (Zhou et al., 2010). Conversely, these compounds also inhibit the pilocarpine-induced expression of the N-methyl-D-aspartate receptor subunit 2B. This inhibition results in reduced synaptic activity and neuronal excitability, thereby playing a significant role in their antiseizure effectiveness (Ndagijimana et al., 2013).

Furthermore, previous studies have identified geissoschizine methyl ether (GM) as a key antiseizure component of *Uncariarhynchophyll* (Miq.) Miq. ex Havil, with its antiseizure effectiveness demonstrated in glutamate-induced seizure models in mice (Jiang et al., 2015). GM’s neuroprotective capabilities have been observed in various glutamate-induced neurotoxicity tests. Its likely mechanisms include the inhibition of inward calcium and sodium currents, the reduction of delayed rectifier potassium currents, the decrease in mitochondrial respiratory functions, and the suppression of reactive oxygen species (ROS) formation (Lai et al., 2014). Research focusing on GM’s effects on neuronal action potential firing, using whole-cell current clamp recordings in cultured murine and rat hippocampal neurons, indicated dose-dependent reductions in firing rates in mouse neurons at concentrations of 1, 3, 10, and 30 μM, with inhibition rates of 45.3% ± 3.7%, 49.6% ± 5.1%, 82.3% ± 1.1%, and 97.8% ± 0.5%, respectively (Xie et al., 2020). In addition to its neuroprotective qualities, GM has been shown to foster myelin repair in developing oligodendrocytes and aid in the restoration of neuronal sheaths in the medial prefrontal cortex. GM also acts as a modulator of serotonergic activity, serving as a partial agonist at 5-HT1A receptors and antagonizing 5-HT2A, 5-HT2C, and 5-HT7 receptors (Nakamura et al., 2018). The compound’s inhibitory effect on acetylcholinesterase also hints at a further neuroprotective advantage, suggesting a potential therapeutic role in Alzheimer’s disease models (Marucci et al., 2021).

Subsequent to its first isolation in 1958 by European scientists from *Vinca minor L* (Feng et al., 2017), vinpocetine marked a significant advancement in plant-based antiseizure therapy. Studies have demonstrated vinpocetine’s ability to enhance memory in animal models, with potential cognitive benefits in humans as well (Meador et al., 2021). Recent clinical evaluations have confirmed vinpocetine’s considerable antiseizure effects, especially in cases of refractory focal epilepsy. Notably, in patients with loss-of-function GABA_A receptor variants, vinpocetine has been effective in reducing seizure occurrence and in improving cognitive and behavioral impairments, thus significantly improving patients’ quality of life (Billakota et al., 2019). For example, a documented case where a patient received 20 mg of vinpocetine thrice daily for 16 months showed a complete cessation of seizures, as confirmed by electroencephalogram findings of substantially reduced epileptiform activities. Alongside seizure control, the patient experienced a significant reduction in associated obsessive-compulsive, anxiety, and depressive symptoms. After 25 months, the dosage of vinpocetine was reduced to 20 mg twice daily with a plan for gradual discontinuation (Gjerulfsen et al., 2023). Continuous cardiac monitoring during the treatment period showed no cardiac function disturbances or changes in routine blood tests, confirming the safety of vinpocetine. Further studies into its mode of action revealed that vinpocetine inhibits sodium and calcium ion conductance, thus stabilizing cellular potentials by limiting ionic influx (El-Sayed et al., 2021). Additionally, vinpocetine has been found to reduce the levels of pro-inflammatory cytokines IL-1β and TNF-α in the rat hippocampus (Gómez et al., 2014).

### 2.2 Antiseizure and neuroprotective abilities of quinoline alkaloids

Quinoline alkaloids, identified by their fundamental quinoline ring structure, constitute a unique group of natural compounds primarily produced through the ortho-aminobenzoic acid pathway (Thongsornkleeb et al., 2022). These alkaloids are widespread in *Hemerocallis fulva* (L.), Rutaceae Juss (often mistakenly called the “Dove family”), and especially in the Cinchona genus of the Rubiaceae Juss family. They are celebrated for their broad range of biological effects. Quinine and camptothecin are particularly noteworthy in this category, having undergone extensive investigation as exemplary quinoline alkaloids. Their principal mechanism of action involves modulating various receptors. Importantly, they have been shown to inhibit NOD-like receptor 3 (NLRP_3_) inflammasome-mediated pathways, reducing inflammatory responses and thus opening new possibilities for anti-inflammatory treatments (Eyal, 2018). Similar to indole alkaloids, quinoline alkaloids reduce epileptic seizures by decreasing NMDA receptor activity and by interacting with altered GABA genes, indicating a diverse approach in managing epilepsy (Shang et al., 2022). Additionally, these alkaloids have a role in suppressing P-glycoprotein (P-gp) expression, which could lower the incidence of epilepsy, thereby paving the way for new research and preventive approaches (Nuthakki et al., 2019). The following sections will delve deeper into quinoline alkaloids, exploring their therapeutic potential in greater detail.

In the early 20th century, pioneering work by Japanese researchers led to the isolation of sinomenine (SN) from the essential oil of *Cymbopogon flexuosus* (Nees ex Steud.) Will. Watson. Initially employed for treating rheumatic conditions, this morphinan alkaloid was subsequently found to possess a wide array of pharmacological properties, including notable antioxidant abilities, and is characterized by a low rate of adverse reactions (Li et al., 2023). Further mechanistic investigations have identified that SN’s neuroprotective effects predominantly stem from its ability to inhibit oxidative stress, neuroinflammation mediated by microglia or astroglia, and neuronal apoptosis (Hong et al., 2022). A controlled experiment using the pentylenetetrazole (PTZ)-induced epilepsy model in rodents demonstrated that SN, administered at doses of 20, 40, 50, and 80 mg/kg, intervened in the seizure initiation process in a dose-responsive manner, effectively reducing seizure intensity and lowering the occurrence of fully developed seizures (Gao et al., 2018). Specifically, a 50 mg/kg dose of SN notably lessened the severity and incidence of status epilepticus (SE), normalized MFS abnormalities in the hippocampus, reduced DNA fragmentation, and preserved neuron density. Concurrently, it significantly restored ROS, MDA, HO-1, and SOD levels, though its impact on GSH levels was not substantial. Additionally, a 50 mg/kg dose of sinomenine partially reversed increases in NF-κB, TLR4, TNF-α, GFAP, and caspase-1 (Ramazi et al., 2020). Moreover, varying SN doses protected hippocampal neurons from PTZ-induced harm and alleviated impairments in spatial learning and memory. These results suggest that SN’s antiseizure and neuroprotective effectiveness is largely driven by its suppression of the NLRP_1_ inflammasome, indicating its potential therapeutic value in epilepsy management (Gao et al., 2018).

In parallel, Xie et al. achieved a breakthrough by extracting neferine from the seeds of *Nelumbo nucifera*, thus enriching the pool of isoquinoline alkaloids renowned for their effectiveness in treating hypertension and arrhythmias (Liu et al., 2023). Methyl-neferine, an alkaloid derived from lotus seed embryos, has come under scrutiny for its prospective neuroprotective role in epilepsy management (Bishayee et al., 2022). In a relevant experiment, rats were pre-treated with methyl-neferines via intraperitoneal injection (i.p.) at doses of 10 and 50 mg/kg, 30 min before administering a KA injection (15 mg/kg, i.p.). This preemptive approach exhibited encouraging outcomes: it delayed the onset of seizures, lessened their intensity, stabilized glutamate concentrations, preserved neuronal health, and enhanced synaptic markers, particularly synaptophysin and postsynaptic density protein 95, in the hippocampal regions of KA-treated rats (Lin T. Y. et al., 2022). Additionally, pre-treatment with methyl-neferine markedly reduced glial cell activation and the consequent inflammatory response in the hippocampus, as indicated by lowered levels of interleukin-1β, interleukin-6, and tumor necrosis factor-α (TNF-α). Significantly, this approach also reduced key markers of the NLRP_3_ inflammasome pathway, including caspase-1 and interleukin-18, in the hippocampi of epileptic rats treated with methyl-neferines (Lin T. Y. et al., 2022). These results collectively indicate the potential of methyl-neferines to alleviate seizure severity, offer neuroprotection, and decrease neuroinflammation in the hippocampus by inhibiting the NLRP_3_ inflammasome and related inflammatory cytokines in KA-induced epilepsy models.

In addition to their role in inhibiting NLRP receptors, quinoline alkaloids also display selectivity in antagonizing NMDA receptors, thus broadening their therapeutic potential. The earliest documented isolation of the quaternary alkaloid berberine, belonging to the isoquinoline subclass, dates back to 1826 when M.-E. Chevalier et al. extracted it from the bark of a *Coptis chinensis* Franch tree (Song et al., 2020). While berberine has long been recognized for its antibacterial properties against organisms like *Staphylococcus aureus* and *Streptococcus* species, it has now been revealed to modulate the neurotransmitter system in a dose-dependent manner, exerting anticonvulsant effects (Gawel et al., 2020). In a study, daily administration of berberine at doses of 25 or 50 mg/kg was performed. The results demonstrated that berberine treatment at a dose of 50 mg/kg in rats microinjected with kainate lowered the incidence of status epilepticus (SE) and spontaneous recurrent seizures. Moreover, it significantly restored hippocampal levels of ROS, glutathione (GSH), nuclear factor (erythroid-derived 2)-like 2, catalase activity, caspase 3 activity, nuclear factor-B, toll-like receptor 4, TNF-α, interleukin-1 beta, and heme oxygenase 1 (Sedaghat et al., 2017). Specifically, berberine attenuates hyperexcitability and abnormal motor patterns in larval models, reducing hypersensitive epileptiform swimming behavior and contributing to the normalization of STX1B gene expression, a critical factor for synaptic function (Jiang et al., 2015). These protective effects are believed to stem from berberine’s antagonism of NMDA receptors, preventing the pathologically excessive activation of extrasynaptic NMDARs, a process implicated in epileptogenesis (Cortes et al., 2015). However, further research is essential to fully elucidate berberine’s capacity as an NMDAR antagonist and its clinical relevance in epilepsy management (Hussain et al., 2018).

Furthermore, the identification and subsequent analysis of erythrosine by-products, specifically (+)-erythravine and (+)-11α-hydroxy-erythravine, derived from *Erythrina mulungu* Mart ex Benth, have broadened the repertoire of compounds exhibiting antiseizure properties (Faggion et al., 2011). Erythrosine, characterized as a quinoline alkaloid with a distinctive benzyl tetrahydroisoquinoline framework, has a well-established history in the management of central nervous system (CNS) disorders, including insomnia and related (Gelfuso et al., 2020). Seizure genesis is often attributed to dysregulations within excitatory and inhibitory neurotransmitter systems. In this context, derivatives of erythrosine reveal their therapeutic potential by modulating acetylcholine-evoked currents, indicating their antagonistic effects on neuronal nicotinic acetylcholine receptors (dos Reis et al., 2021). The antiseizure efficacy of both (+)-erythravine and (+)-11α-hydroxy-erythravine is noteworthy in various seizure models induced by phenyltetrazolium, kainic acid, bicuculline, and NMDA, implying a broad spectrum of activity (Dakic et al., 2016). Building upon this foundation, *in vivo* experiments demonstrate that both compounds, administered at dosages of 1.23 μg/μL, are efficiently absorbed through the gastrointestinal tract and possess the capacity to penetrate the BBB, a pivotal pharmacokinetic attribute for CNS-targeted therapeutics (Gelfuso et al., 2020). This permeability underscores the therapeutic relevance of these compounds and propels the advancement of novel antiseizure medications (Gelfuso et al., 2020).

Expanding our perspective, quinoline alkaloids exhibit diverse pharmacological actions that extend beyond NMDA receptor antagonism, encompassing effects on GABAergic systems. Tetrahydropalmatine, a benzylisoquinoline alkaloid derived from *Corydalis yanhusuo* W. T. Wang, has traditionally been employed to enhance blood flow, alleviate stasis (Oh et al., 2010). Investigations into its mechanistic effects on epilepsy models reveal a reduction in dopamine secretion, along with an enhancement of GABAergic and cholinergic receptor functionalities (Wu et al., 2015). Notably, experimental intraperitoneal injections of Tetrahydropalmatine at doses of 10 mg/kg or 15 mg/kg in rats demonstrated that pretreatment with Tetrahydropalmatine nearly completely eliminated the Picrotoxin-induced increase in dopamine release in the amygdala (Chang and Lin, 2001). Such modulation leads to the suppression of seizure initiation, highlighting the potent antiseizure properties of corydaline. Additionally, in the 1950s, isoquinoline alkaloids like montanine, derived from *Hippeastrum vittatum* (L’Hér.) Herb, initially gained attention for their anticancer properties, particularly their ability to inhibit malignant cell proliferation. Subsequent research, however, has unveiled significant anxiolytic and antiseizure benefits associated with montanine (Farinon et al., 2017). Experimental data elucidates its role in preventing convulsions by acting on the GABA_A_ receptor system. This modulation not only optimizes GABA receptor conductance but also corrects aberrant neurotransmitter distribution, offering a promising therapeutic approach for managing epilepsy.

Furthermore, the therapeutic potential of quinoline alkaloids extends beyond the modulation of neurotransmitter receptors, encompassing the inhibition of protein expression and regulation of ion channels (Plazas et al., 2022). Tetrandrine (TTD), a dibenzylisoquinoline compound sourced from *Stephania tetrandra* S. Moore, has traditionally been indicated for the management of hypertension (Zhang Z. X. et al., 2018). However, recent evidence suggests the utility of TTD in epilepsy, highlighting its capacity to reduce the expression of P-gp, thus enhancing the efficacy of AED (Chen et al., 2015). In a study, epileptic rats receiving intraperitoneal TTD at a dose of 30 mg/kg displayed significant behavioral improvements assessed using Racine’s seizure scale over a 2-week observation period, in contrast to the saline-treated control group (Chen et al., 2015). While these outcomes are promising, further investigation is required to comprehensively evaluate TTD’s therapeutic profile and safety for the treatment of epilepsy.

Similarly, quinidine, another alkaloid extracted from *Cinchona ledgeriana* (Howard) Moens ex Trim, is well-known for its antiarrhythmic properties. Recent research has shifted its focus towards the application of quinidine in precision medicine for seizure disorders associated with KCNT_1_ gene mutations (Yoshitomi et al., 2019). *In vitro* studies using *Xenopus laevis* oocytes have revealed that quinidine exerts a concentration-dependent correction of the abnormal increase in potassium channel activity caused by KCNT_1_ mutations. These mutations are implicated in conditions such as autosomal dominant nocturnal frontal lobe epilepsy and severe infantile-onset epileptic encephalopathy. Quinidine’s ability to modulate potassium channel function supports its role in reducing neuronal hyperexcitability and consequent seizure manifestations.

### 2.3 Modulation of neuronal excitability by terpenoid alkaloids for seizure control

Terpenoid alkaloids constitute a unique class of compounds characterized by the fusion of nitrogenous groups with terpenoid backbones. They can be further classified based on the terpenoid component into monoterpenoids, sesquiterpenoids, diterpenoids, and triterpenoids (Huang et al., 2022). Nantenine, a terpenoid alkaloid originally derived from *Nandina domestica* Thunb (Qin et al., 2021), is a notable example of this class used clinically for its sedative and antiseizure properties. Research suggests that nantenine modulates calcium-mediated signaling, reducing the hyperexcitability of neuronal cells by regulating the influx of calcium. This presents a potential therapeutic approach for epilepsy by stabilizing neuronal excitability and mitigating seizures (Steinlein, 2014). On the other hand, aconitine, another noteworthy terpenoid alkaloid, is categorized as a neurotoxic diterpene due to its diester configuration. It is known for its stimulatory effect on the vagus nerve and has been observed to interact with voltage-gated sodium channels, which play a crucial role in regulating neuronal excitability (Ono et al., 2013). Research by Ameri (1998) has examined the central nervous system effects of aconitine, revealing its selective action on sodium channels and its potential as an antiseizure agent in rat hippocampal slices. However, further validation is needed to establish the efficacy and safety of aconitine for epilepsy treatment.

### 2.4 Antiseizure effects of pyridine alkaloids: GABA receptor targeting and antioxidant properties

Pyridine alkaloids, synthesized from lysine and branching into pyridine or piperidine derivatives, are characterized by their streamlined and minimalistic structures (Frolov and Vereshchagin, 2023). They can be generally categorized into two subsets: uncomplicated pyridines and bis-piperidines (Ma et al., 2014). Notable examples within this alkaloid family include nicotine, found in tobacco, and piperine, present in black pepper. These compounds primarily exert antiseizure effects by targeting and inhibiting mutations within genes that encode GABA receptors (Lin et al., 2020).

Piperine, a pyridine alkaloid derived from *Piper longum L.*, exerts a wide-ranging anticonvulsant influence (Ren, 2019). Beyond seizure mitigation, it finds clinical applications in analgesia, anxiolysis, and lipid regulation. Mechanistically, piperine modulates the central neurotransmitter milieu, particularly by increasing cortical and hippocampal 5-hydroxytryptamine (serotonin) and GABA levels. This modulation correlates with the attenuation of epileptic tonic-clonic seizures (da Cruz et al., 2013). Notably, piperine (administered at 10 mg/kg) delays the onset of tonic-clonic convulsions and reduces associated mortality in the pentetrazol trial. Furthermore, anticonvulsant doses of piperine delay the onset of strychnine, picrotoxin, and BAYK-8644 tonic-clonic seizures. Electrophysiological evidence supports its role in inhibiting sodium and calcium channel functions (Mishra et al., 2015). However, it is essential to note that while these findings are promising, the research on piperine’s antiseizure utility remains preliminary, necessitating extensive longitudinal studies to uncover its full clinical potential.

Simultaneously, the compound boldine, extracted from the root of the *Litsea cubeba* (Lour.) Pers. and categorized as an aporphine isoquinoline alkaloid, has garnered attention for its utility in antioxidant regimens (de Souza et al., 2022). The investigative efforts have spotlighted boldine’s seizure-preventive capacity in murine models, challenged with convulsions induced by hydrazine and indole dioxides. Administered intraperitoneally, boldine demonstrated dose-dependent efficacy in obstructing PTZ-induced clonic and myoclonic seizures and curtailed the seizure duration in models of electric shock-induced convulsions (Akotkar et al., 2023). Its modulatory effects on GABA receptors confer neuroprotection, particularly against dysfunctional GABA receptor mutations. Furthermore, boldine’s long-term administration has been associated with augmented levels of glutathione and superoxide dismutase, reflecting its intrinsic antioxidant capabilities (Akotkar et al., 2023). Experimental data prove, Acute administration of non-effective dose of boldine (10 mg/kg) and vitamin C (50 mg/kg) induced anti-convulsing effects of boldine on seizure threshold (Moezi et al., 2019). Its free radical neutralizing activity, especially against hydroxyl radicals, positions boldine as a candidate of considerable interest for epilepsy treatment strategies. These viewpoints collectively expand the treatment landscape for alkaloids in epilepsy management (Moezi et al., 2019). Pyridine alkaloids can also exert their effects by inhibiting synaptic transmission, and tetramethylpyrazine, a pyridine alkaloid derived from Conioselinum anthriscoides (H. Boissieu) Pimenov, is one such compound recognized for its antiseizure properties in clinical settings (Lin J. et al., 2022). The mechanistic profile of tetramethylpyrazine is complex and diverse: it modulates both neurotransmitters and inflammatory cytokines, reducing neuronal hyperactivity and providing neuroprotection (Meng et al., 2021). Additionally, it downregulates P-gp expression in cerebral tissues, mitigating impediments to BBB permeability. This enhancement of hemorheological attributes counters stress-related damage and modulates immune responses (Qian et al., 2023). In a study, mice treated with TMP (20 and 50 mg/kg, i.p.) remained in stage 1 of epileptic progression for an extended period, requiring additional stimulation to induce stages 2–5 epileptic phenotypes. TMP (50 mg/kg) also inhibited 6 Hz corneal kindling progression (Jin et al., 2019). Experimentally, tetramethylpyrazine’s ability to adjust glutamate and GABA synthesis in the brain is noteworthy. It suppresses glutamate synthesis while augmenting GABA production, fostering equilibrium between excitatory and inhibitory neurotransmission. This dampens neuronal hyperexcitability and ultimately inhibits seizure activity (Danduga et al., 2018). Moreover, tetramethylpyrazine plays a role in synaptic remodeling by upregulating neuroprotective molecules and curtailing the expression of apoptosis-related factors, contributing to its neuroprotective and antiseizure effects (Lin T. Y. et al., 2022). Despite these promising attributes, the intricate pathways and the full spectrum of tetramethylpyrazine’s impact necessitate further elucidation.

Notably, the pyridine derivative oxysophocarpine (OSC) has come to prominence for its compelling anticonvulsant and neuroprotective attributes (Xu et al., 2013). In murine models, specifically adult males subjected to rutaecarpine-induced convulsions, OSC administration has been shown to significantly delay the onset of both initial convulsive activity and sustained status epilepticus, as evidenced by electroencephalographic assessment. Moreover, subsequent to administration of 40 mg/kg and 80 mg/kg doses, a marked suppression of epileptiform activity was observed. Histological analyses, including Nissl and fluoro-Jade B staining, revealed OSC’s capacity to mitigate hippocampal neuronal attrition and degradation (Liu et al., 2017). Complementary bioassays indicated a decrease in malondialdehyde levels and an increase in enzymatic activities associated with antioxidative defenses, glutathione peroxidase, and catalase, suggesting a reduction in oxidative stress. Further molecular studies, using Western blot technique, clarified that the expression of Bax and Caspase-3 decreased, while the expression of Bcl-2 augmented, indicating that OSC downregulated neuronal apoptosis (Liu et al., 2017).

### 2.5 Applications of ephedritosisne and pinellia alkaloids in managing epilepsy

Ephedrine, an alkaloid denoted chemically as C_10_H_15_NO, transcends its classification as a Class I vulnerable chemical due to its extensive clinical utility. It is renowned primarily for staving off bronchial asthma attacks and ameliorating mild asthmatic symptoms (Gad et al., 2021). Recent studies have uncovered additional pharmacological properties of ephedrine, specifically an anti-epileptic activity derived from extracts of *Herba Ephedrae* (Zhang B. M. et al., 2018). This novel insight broadens the medicinal applications of ephedrine and herald’s new prospects for epilepsy management.

Pinellia total alkaloids (PTA), sourced from the *Pinellia ternate* (Thunb.), have been empirically validated for quelling seizure manifestations in maximal electroconvulsive and penicillin-induced ignition models (Bai et al., 2022). In a structured experimental framework, 91 male Sprague-Dawley rats were allocated into control and epileptic cohorts, with the latter induced into sustained epilepsy via hirsute fruit rutabaga administration (Deng et al., 2020). Post-SE survivors were further segmented into groups receiving either no treatment, topiramate, high-dose PTA (800 mg/kg), or low-dose PTA (400 mg/kg), with daily gavage over a fortnight. Aftertreatment, spontaneous recurrent seizures were observed over a week, followed by hippocampal tissue collection for GABA quantification via enzyme-linked immunosorbent assay. Results revealed that PTA modulated GABA levels and influenced the expression of GAD65, GAT-1, GABA-T, and GABA_A_R subunits α4, α5, γ2, and δ, suggesting a mechanistic basis for its antiseizure effect against epilepsy prompted by Hirsutine. The elevation in GABA concentrations and upregulation of GABA_A_ receptor mRNA in the cerebellum underscore PTA’s enhancement of the inhibitory GABAergic system function (Deng et al., 2020; Richardson et al., 2024). These findings illuminate the therapeutic potential of PTA in epilepsy treatment and enrich the discourse on the pathogenesis and therapeutic approaches to the condition. Nevertheless, the translation of PTA to a clinical antiseizure modality demands further validation to ascertain its long-term efficacy and safety.

In brief, we explore the antiepileptic properties of five distinct alkaloids, which mitigate epileptic disruptions by various mechanisms: decreasing excessive Ca^2^⁺ ion flux due to hyperactive NMDA receptors, sustaining GABAergic signaling inhibition, modulating voltage-gated ion channels (Na⁺, Ca^2^⁺, and K⁺), and acting as agonists at the 5-HT1A receptor while antagonizing the 5-HT2A receptor. Despite their demonstrated efficacy in alleviating epilepsy, these alkaloids face several challenges, including poor solubility and bioavailability, exemplified by compounds such as piperine, as well as insufficient lipid solubility, which hinders berberine’s ability to effectively penetrate the BBB (Quijia et al., 2021). These limitations can impact the unique therapeutic potential of alkaloids in epilepsy management. In the subsequent section, we will elaborate on effective and innovative strategies to overcome issues like the low bioavailability of these compounds.

## 3 Alkaloid therapy for epilepsy: challenges and opportunities

### 3.1 Exploration of methods to improve low bioavailability of alkaloids

We have identified various strategies to improve the low bioavailability of alkaloids. For instance, Huang et al. (2016) synthesized a series of novel C-9-O substituted berberine derivatives and evaluated their anti-inflammatory effects both *in vitro* and *in vivo*. These novel synthetic berberine derivatives demonstrated significantly higher inhibitory activity on NO, TNF-α, and IL-6 release, along with enhanced bioavailability compared to berberine. Similarly, Zhang et al. (2020) synthesized a berberine derivative, hydrophobic ethyl-2-(9-dimethoxyberberine bromide-9-yl) hydroxy acetate, which exhibited an enhanced anticonvulsant effect directly linked to improved bioavailability.

**FIGURE 4:**
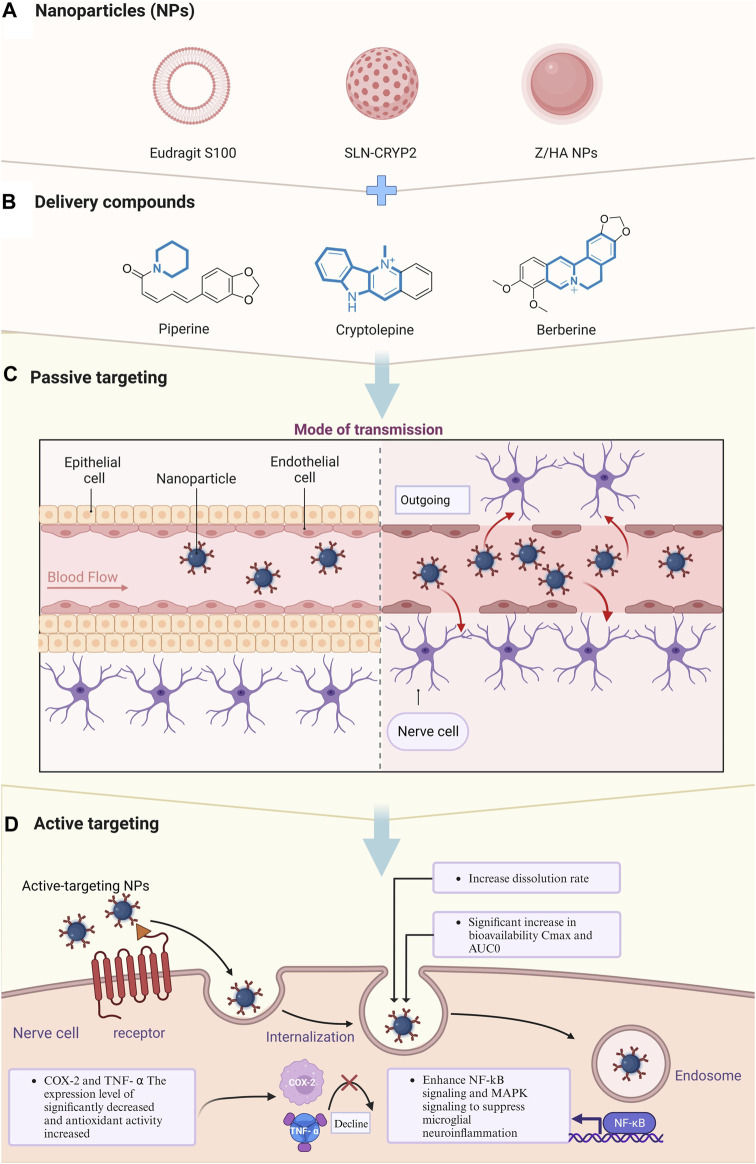
Delivery of natural alkaloids to nerve cells via nanomaterial delivery systems for therapeutic effects. **(A)** Types of nanomaterials encapsulating small molecule compounds. **(B)** Structural formulas of improved epilepsy therapeutic compounds utilizing nanomaterial encapsulation. **(C)** Transport of nanomolecules from blood vessels into nerve cells after crossing endothelial barriers. **(D)** Intracellular entry of nanomolecules and enhancement of physicochemical properties of drugs.

In addition to traditional chemical modifications to enhance physicochemical properties, emerging nanotechnologies offer a transformative direction. For example, Shilo et al. (2015) highlighted nanoparticles as promising drug delivery agents capable of crossing the BBB in 2015. Nanoparticles have garnered significant scientific interest due to their versatile properties, making them suitable for various biological and chemical applications (van Rooy et al., 2011). Nanoparticle-based drug delivery systems show promise in overcoming bioavailability challenges by enhancing drug solubility, stability, and permeability through the BBB (Duan et al., 2023). Our forthcoming investigation will focus on optimizing alkaloid delivery through nanoparticle technology. This innovative approach aims to maximize therapeutic efficacy by enabling targeted and controlled release of alkaloids, presenting a potential breakthrough in epilepsy treatment (Figure 4; Table 2).

**TABLE 2:** Enhancement of natural alkaloids’ properties via nanomaterial delivery systems.

Compounds	Particle size (nm)	Maximum release rate (%)	BBB permeability	Hepatorenal toxicity	Nanoparticle advantage	Ref.
*Piperine*	130.2 ± 1.57	64.7 ± 3.0	*P* = 3.77E - 14	**—**	Increased dissolution rate Increased bioavailability C_max_ and AUC0 Increased liposolubility and makes it easier to penetrate the BBB	Anissian et al. (2018), Ren et al. (2019), Zhu et al. (2020)
*Cryptolepine*	152.4 ± 16.3	83.44 ± 3.16	*Pe* = 10.81 ± 0.19×10^−6^ cm/s	**—**	Increased dissolution rate Enhanced NF-κB signaling and MAPK signaling to suppress microglial neuroinflammation	Mante et al. (2021)
*Berberine*	297.2 ± 1.86	84.23 ± 2.34	*C* = 40.29 ± 28.59 ng/g	*p* > 0.05	Increased dissolution rate Decreased COX-2 and TNF-α expression level and increased antioxidant activity	El-Nahas et al. (2023), Saha et al. (2023)

### 3.2 Enhancing alkaloid efficacy in epilepsy treatment using nanoparticle delivery systems

In 2019, Ren et al. (2019) has presented a pioneering study that effectively enhances the solubility of piperine, an alkaloid with antiseizure potential, through innovative nanotechnology. By reformulating piperine into a nanoparticle suspension using a nanoprecipitation technique, they aimed to amplify its oral bioavailability, thereby potentiating its development as an antiseizure agent. Employing transmission electron microscopy for meticulous characterization, they confirmed the formation of uniform, spherical nanoparticles with a narrow size distribution and high encapsulation efficiency (Zhu et al., 2020). Piperine was first used for optimization in the nano field. Remarkably, the nanoformulated piperine exhibited a significant boost in dissolution rates, with a threefold increase in the 24-hour cumulative drug release, compared to its conventional counterpart. When administered to rats at a dosage of 3.5 mg/kg, the nanoformulation achieved a 2.7-fold rise in oral bioavailability and demonstrated a marked 16-fold escalation in brain concentration within 10 h post-administration (Ren et al., 2019). The efficacy of these piperine nanoparticles was further validated *in vivo*, where they afforded robust protection against pentylenetetrazole-induced seizures in zebrafish and mouse models, showcasing their potential as a formidable strategy in epilepsy management (Anissian et al., 2018).

Subsequently, in 2021, Riscilla et al. innovated a solid lipid nanoparticle delivery system for cryptolepine, denoted as SLN-CRYP, that substantially improved the compound’s capacity to traverse the BBB. This advancement not only facilitated enhanced drug-receptor interaction but also increased the drug’s permeability into the brain. They employed a PTZ-induced epileptiform behavioral model in zebrafish to quantify the antiseizure efficacy of SLN-CRYP, testing dosages of 2.5 and 5 mg/kg (Mante et al., 2021). A rigorous examination into SLN-CRYP’s pharmacokinetics affirmed a marked improvement in BBB permeability. Clinically significant findings emerged from the treatment outcomes: both tested dosages of SLN-CRYP substantially decreased the average seizure scores and markedly extended the onset of seizure episodes. Furthermore, receptor binding assays revealed that cryptolepine displayed inhibitory effects on human voltage-gated calcium channels (Cav1.2), H1 receptors, peripheral benzodiazepine receptors, and σ2 receptors, albeit with relatively modest affinity, indicating a lesser antagonistic impact on Cav1.2 and σ2 receptors compared to known antagonists nifedipine and haloperidol, respectively (Mante et al., 2021). This underlines the therapeutic relevance of SLN-CRYP as a promising candidate for epilepsy treatment, with potential for further investigation and development.

Recently, in exploring the delivery system of AED, Amira et al. has made a notable advance in the pharmacokinetic enhancement of antiseizure agents by formulating composite nanoparticles for the augmented cerebral absorption of berberine. These nanoparticles, designated B2, have been shown to significantly ameliorate the manifestations of persistent status epilepticus in rats, stimulating composite nanoparticles for the augmented cerebral absorption of berberine. These nanoparticles, designated B2, have been shown to significantly ameliorate the manifestations of persistent status epilepticus in rats, a condition notoriously challenging to manage with traditional berberine suspensions (El-Nahas et al., 2023). This innovation suggests that the novel nanoparticle construct could markedly improve the clinical management of epilepsy. Moreover, an analysis of hippocampal tissue from B2-treated rats revealed diminished neurodegeneration, which was further underscored by a pronounced decrease in pro-inflammatory markers COX-2 and TNF-α, alongside an increase in antioxidative measures. These results underscore the dual therapeutic benefit of B2 mitigating the pathological sequelae of epilepsy and concurrently promoting neurohealth. Crucially, safety evaluations of the nanoparticle system yielded no evidence of toxicity or behavioral modification, affirming the biocompatibility of B2 (El-Nahas et al., 2023). This finding substantiates the nanoparticle system’s potential as a safe vehicle for delivering AED, holding promise for future clinical translation (Saha et al., 2023).

## 4 Conclusion and outlook

This review begins by examining the diverse neuropharmacological activities of natural alkaloids that position them as potential treatments for epilepsy. Natural alkaloids confer therapeutic benefits by modulating synaptic communication, reestablishing neuronal equilibrium, and mitigating neuroinflammation, mechanisms critical for the control of epileptic seizures (Sharifi-Rad et al., 2021). Salient therapeutic actions include the potentiation of GABAergic neurotransmission, the attenuation of excitatory glutamatergic activity, notably at NMDA receptors, and the suppression of pro-inflammatory cascades (Mathie, 2010). Collectively, these actions bolster the candidacy of natural alkaloids as efficacious agents in the management of epilepsy (Figure 3). Despite promising laboratory studies, the application of these alkaloids in clinical settings is still in its early stages, with a majority of findings confined to animal studies and a dearth of human clinical trial data. Consequently, further empirical scrutiny into the toxicological profiles and the safety of natural alkaloids is imperative. Prospective research trajectories should pivot on the explication of underlying molecular pathways, the longitudinal assessment of efficacy and safety in controlled human studies, thereby bridging the gap towards the incorporation of these alkaloids into mainstream epilepsy therapeutics (Suzuki et al., 2011).

The therapeutic potential of alkaloids in epilepsy, however promising, is compromised by their limited solubility and bioavailability, which pose substantial barriers to their clinical deployment. Addressing these limitations necessitates technological refinements aimed at bolstering the stability and efficacy of these compounds. Strategies extend beyond nanodelivery systems to include the intricate re-engineering of chemical structures, an area burgeoning with potential. A case in point is the innovation surrounding zembrin, an alkaloid-rich extract utilized in epilepsy treatment. This botanical compound is composed of mesembrine, mesembranol, mesembrenol, and mesembrenone (Dimpfel et al., 2018). Through meticulous structural modifications, specifically, substituting the carbonyl group at the C6 position with a hydroxyl moiety in mesembranol and mesembrenol, researchers have unearthed new structure-activity relationships. The resultant analogs target and mitigate AMPA receptor-mediated excitatory transmission, thereby enhancing their antiseizure profile. Clinical investigations have substantiated the efficacy of these modified alkaloids, with dosages of 5 and 10 mg/kg mirroring the therapeutic outcomes of a 50 mg/kg standard dose. Such findings elevate mesembranol and mesembrenol to the status of pivotal chemical forerunners in the quest to forge novel antiseizure agents, potentially invigorating the entire epilepsy treatment landscape (Dimpfel et al., 2018).

In parallel with enhancing alkaloid bioavailability, it is imperative to rigorously assess their potential toxicological implications to preempt any irreversible physiological detriment. Crucial to their advancement as treatments is a thorough examination of their safety, a step that is currently lagging behind other research areas. For instance, certain isoquinoline alkaloids like berberine exhibit an affinity for DNA, precipitating toxicological responses (Liu et al., 2019). Moreover, organic amine alkaloids, exemplified by ephedrine, may induce cardiotoxic sequelae, such as hypertension and palpitations (Neumann et al., 2023). Given these risks, vigilance in monitoring the toxicological profile of alkaloids used in epilepsy treatment is essential during experimental trials. Yet, the scope of research addressing the toxicology of potential antiseizure alkaloids remains disappointingly narrow. This gap in the literature underscores the necessity for intensified research into the toxic effects of alkaloids, which is critical for elucidating their safety and ensuring informed therapeutic use.

The advancement of medical science calls for a concerted effort to refine current alkaloid-based treatments and to pioneer new avenues for epilepsy management. While discussing the advances in epilepsy treatment, it is pertinent to consider the unique position of alkaloids within this broader context. Notably, the second-generation AED levetiracetam has demonstrated superior seizure control by precisely modulating neurotransmitter release through synaptic vesicle glycoprotein 2A, representing a significant advancement towards precision medicine (Celdran de Castro et al., 2023). Additionally, the third-generation AED perampanel has brought fresh perspectives by antagonizing AMPA receptor-mediated excitatory transmission (Perversi et al., 2023). Using mTOR as a target, the novel therapeutic agent everolimus improves the understanding of the pharmacological framework of epilepsy isolated from Auvin and Baulac (2023). These emergent drugs and their mechanisms not only furnish novel therapeutic modalities but also unveil yet unexplored therapeutic strategies and potential drug targets (Jain et al., 2022). Moreover, the prospect of alkaloids augmenting combination therapies marries traditional herbal insights with contemporary pharmacology, representing a compelling research frontier. Combining treatments such as Nobiletin with clonazepam, or naringenin with phenytoin, has yielded remarkable clinical outcomes, expanded the repertoire of epilepsy treatment strategies and enhanced therapeutic efficacy. While alkaloids currently represent a minor component of these combination therapies, the deepening insight into their pharmacodynamics and escalating research funding justify a confident forecast: In the not-too-distant future, the integration of alkaloids with other therapeutic agents is poised to emerge as a pivotal strategy in epilepsy management. This evolution will undoubtedly offer more efficacious, tailored treatment options for individuals afflicted with this neurological condition.

In conclusion, the promise of alkaloids in epilepsy treatment is contingent upon dedicated research efforts to refine their delivery mechanisms and safety profiles. Future research endeavors are anticipated to address these obstacles, enhancing the solubility, bioavailability, and safety profile of alkaloid-based therapies. Advancements in pharmacological research and drug development processes are poised to deliver more efficacious and safer treatment alternatives to individuals living with epilepsy, heralding a new epoch in the management of this complex neurological disorder. The culmination of these efforts will be a paradigm shift in epilepsy therapeutics, driven by a commitment to innovation and a deepened understanding of plant-derived pharmacology.

## Abbreviations

ASM, antiseizure medications; BBB, blood-brain barrier; CNS, central nervous system; GM, geissoschizine methyl ether; KA, kainic acid; MAPK, mitogen-activated protein kinase; NLRP_3_, nod-like receptor 3; OSC, oxysophocarpine; P-gp, p-glycoprotein; PTA, pinellia total alkaloids; PTZ, pentylenetetrazole; ROS, reactive oxygen species; RP, rhynchophylline; SE, status epilepticus; SN, sinomenine; TNF-α, tumor necrosis factor-α; TTD, tetrandrine.
